# OphthoEvidence Report: Comparative effects of treatments for geographic atrophy secondary to age-related macular degeneration: A protocol for a living systematic review and network meta-analysis

**DOI:** 10.1371/journal.pone.0358699

**Published:** 2026-09-30

**Authors:** Maryam Ghadimi, Housne Begum, Dena Zeraatkar, Jinhui Ma, Sobha Sivaprasad, Enrico Borrelli, Peter Kaiser, David H. Steel, Tien Yin Wong, Robyn Guymer, Charles C. Wykoff, Varun Chaudhary

**Affiliations:** 1 Division of Ophthalmology, Department of Surgery, McMaster University, Hamilton, Ontario, Canada; 2 Thomas Kevill Advanced Vitreoretinal Imaging (AVI) Lab, St Joseph’s Healthcare, Hamilton, Ontario, Canada; 3 Department of Health Research Methods, Evidence, and Impact, McMaster University, Hamilton, Ontario, Canada; 4 Department of Anesthesia, McMaster University, Hamilton, Ontario, Canada; 5 NIHR Biomedical Research Centre at Moorfields Eye Hospital and UCL Institute of Ophthalmology, London, United Kingdom; 6 Department of Surgical Sciences, University of Turin, Turin, Italy; 7 Department of Ophthalmology, “City of Health and Science” Hospital, Turin, Italy; 8 Cole Eye Institute, Cleveland Clinic, Euclid Avenue, Cleveland, Ohio, United States of America; 9 Sunderland Eye Infirmary, National Health Service, Sunderland, United Kingdom; 10 Biosciences Institute, Newcastle University, Newcastle Upon Tyne, United Kingdom; 11 Singapore Eye Research Institute, Singapore National Eye Centre, Singapore Singapore; 12 Beijing Visual Science and Translational Eye Research Institute, School of Clinical Medicine, Beijing Tsinghua Changgung Hospital, Tsinghua Medicine, Tsinghua University, Beijing, China; 13 Center for Eye Research Australia, Royal Victorian Eye and Ear Hospital, The University of Melbourne, Melbourne, Australia; 14 Retina Consultants of Texas, Retina Consultants of America, Blanton Eye Institute, Houston Methodist Hospital, Houston, Texas, United States of America; Yokohama City University, JAPAN

## Abstract

**Background:**

Geographic atrophy (GA), an advanced form of age-related macular degeneration (AMD), leads to legal blindness in approximately 16% of affected individuals within 6 years. Numerous trials have evaluated interventions to slow GA progression, and many studies are ongoing. However, the comparative benefits and harms of treatments within this rapidly evolving therapeutic landscape have not yet been systematically synthesized.

**Objectives:**

To present a protocol for a living systematic review and network meta-analysis (NMA) addressing the effects of available interventions for the management of GA.

**Methods:**

We systematically searched Medline, EMBASE, Cochrane CENTRAL, Web of Science, and ClinicalTrials.gov from inception for randomized controlled trials comparing any pharmacologic or non-pharmacologic treatment versus another, placebo, sham, or standard care in adults with unilateral or bilateral GA secondary to AMD. Paired reviewers will independently screen studies, extract data, and assess risk of bias. Outcomes of interest include best-corrected visual acuity (BCVA), untransformed and square root-transformed total GA lesion area, vision-related quality of life, low luminance BCVA, reading speed, mean macular sensitivity, adverse events leading to discontinuation, macular neovascularization, need for rescue treatment, ischemic optic neuropathy, serious ocular adverse events, and serious non-ocular adverse events. For each outcome, if feasible, we will conduct frequentist random-effects NMAs. We will assess the certainty of evidence and interpret findings using the GRADE approach. To maintain this review as a living systematic review, we will conduct updates every six months.

**Discussion:**

This review will provide a comprehensive, trustworthy, and up-to-date summary of evidence on treatments for GA secondary to AMD.

**Registration:**

Open Science Framework (https://doi.org/10.17605/OSF.IO/W4JHR).

## Introduction

Age-related macular degeneration (AMD) is a progressive chorio-retinal disease and one of the leading causes of vision loss among older adults worldwide [1]. AMD advances through early, intermediate, and late stages, with clinical manifestations ranging from subtle, asymptomatic changes to severe visual impairment [2]. Geographic atrophy (GA), also known as dry AMD, is a form of late AMD and is characterized by loss of retinal pigment epithelial cells, photoreceptors, and choriocapillaris [3,4]. GA affects an estimated 5 million individuals globally [5] and approximately 16% of cases progress to legal blindness within 6 years [6]. Progressive vision deterioration results in substantial functional impairment, including loss of independence, increased risk of falls, and higher rates of depression and cognitive decline, underscoring the considerable public health burden of the disease [7,8].

Currently, complement inhibition represents the only approved pharmacologic treatment for GA, with pegcetacoplan and avacincaptad pegol approved in the United States; however, none are currently approved for GA in Canada or the European Union [9–11]. Several other interventions have been tested in randomized controlled trials (RCTs) with the aim of slowing GA progression, and many trials are ongoing [12]. These interventions include regulators of the alternative complement pathway (e.g., inhibitors of factor B or factor D), visual cycle modulators, mitochondrial function modulators, antioxidants, nutritional supplements, gene or cell-based therapy, electrical stimulation, photobiomodulation, surgical interventions, and emerging investigational therapies.

Although approved therapies exist, important questions remain regarding the comparative effects of available interventions. Summarizing the benefits and harms of all interventions through a systematic review of available evidence can help address this gap and support evidence-based decision-making [13]. Previous systematic reviews were limited in scope, focusing on specific classes of interventions, failed to include all patient-important outcomes, or to assess the certainty of evidence [14–17]. To address current limitations and account for the rapidly evolving therapeutic landscape of GA, we present a protocol for a living systematic review and network meta-analysis, addressing efficacy and safety of all pharmacologic and non-pharmacologic interventions in adults with GA secondary to AMD.

## Materials and methods

Our protocol adhered to the Preferred Reporting Items for Systematic Reviews and Meta-Analysis Protocols (PRISMA-P) guidelines (S1 Table) [18]. We registered this review in Open Science Framework [19] and will report our systematic review in accordance with the Preferred Reporting Items for Systematic Reviews and Meta-Analyses (PRISMA) with network meta-analysis extension [20,21]. Table 1 presents the status and timeline of the review.

**Table 1:** Stages of the systematic review and timeline.

Stages of the review at the time of submission	Started	Anticipated completion date
Development of search strategy	Yes	Completed
Systematic search	Yes	Completed
Screening records	Yes	Completed
Data extraction	No	August 15, 2026
Risk of bias assessment	No	August 15, 2026
Data synthesis and analysis	No	September 15, 2026
Assessment of certainty of evidence	No	September 30, 2026
Interpretation of findings	No	October 10, 2026
Preparation of manuscript	No	October 30, 2026

### Eligibility criteria

We will include randomized controlled trials (RCTs) evaluating any approved or investigational pharmacologic or non-pharmacologic treatment or nutritional supplement compared with an alternative treatment, supplement, placebo, sham, or standard care in patients aged 50 years or older with a clinical diagnosis of unilateral or bilateral GA secondary to AMD confirmed by an ophthalmic imaging modality (such as fundus autofluorescence (FAF), optical coherence tomography (OCT), colour fundus photography (CFP), or near-infrared reflectance), demonstrating uni- or multifocal atrophic lesion of any size, with subfoveal (i.e., central) or non-subfoveal (i.e., non-central) involvement at baseline. We will include trials in which either patients or individual eyes were randomized. We will not restrict eligibility criteria based on the imaging technique used for the diagnosis of GA. However, subgroup analysis will test how different diagnostic imaging techniques influence the effects of interventions.

For trials including participants with GA alongside other AMD stages (e.g., drusen abnormality, pre-atrophic AMD (e.g., nascent or incipient GA), or neovascular AMD), we will include them if at least 80% of participants had GA in the study eye or if data are reported separately for the GA subgroup.

We will not restrict eligibility criteria based on the type of interventions used for the treatment of GA. Trials that evaluated low vision rehabilitation strategies, including vision aid devices and assistive technologies, will not be eligible. We will include trials regardless of language, year of publication, country of origin, and publication status. Due to the degenerative nature of GA and the potential for carry-over effects with treatments, we will exclude cross-over trials [22].

### Search strategy and study selection

An experienced medical librarian developed specific search strategies for Medline, EMBASE, Cochrane CENTRAL, Web of Science, and ClinicalTrials.gov from inception (S2 File). We will supplement our search with backward and forward citation searching using citationchaser [23]. We will also review the list of studies included in relevant systematic reviews for potentially eligible studies not captured by our search. Following training and calibration exercises, using Covidence [24], pairs of reviewers independently screened titles and abstracts of retrieved citations, and subsequently full texts of potentially eligible studies. Reviewers resolved discrepancies by discussion and, if necessary, by consultation with a third reviewer.

### Data extraction

Following training and calibration exercises, using standardized pilot-tested data extraction forms, pairs of reviewers will independently extract data from included studies. Reviewers will resolve discrepancies by discussion and, if necessary, by consultation with a third reviewer. From each eligible study, we will extract information on study characteristics (e.g., year, country of origin, sources of funding), patient characteristics (e.g., age, sex, presence of neovascular AMD in the study eye, presence of early/intermediate or neovascular AMD in the fellow eye, baseline visual acuity), GA lesion characteristics (e.g., diagnostic imaging modality, proportion with bilateral involvement, proportion with subfoveal involvement, distance from fovea for non-subfoveal lesions, lesion size at baseline, proportion with multifocal lesions, proportion with perilesional hyperautofluorescence), details of intervention and comparator (e.g., intervention type, dosing regimen, duration of treatment), rescue therapy, and outcomes of interest.

We classified change in best-corrected visual acuity (BCVA), change in untransformed total GA lesion area, and change in square root-transformed total GA lesion area, all measured in the study eye, as critical outcomes. We will extract the GA lesion area measured by FAF, if not reported, in the preferred order: OCT, near-infrared reflectance, and CFP. We will prioritize GA area adjudicated by a central reading centre over GA area reported from local reading centres. The important outcomes include change in vision-related quality of life based on 25-item Visual Function Questionnaire [25], change in low luminance BCVA (LL-BCVA, study eye), change in reading speed (study eye), change in mean macular sensitivity measured by microperimetry (study eye), any adverse events leading to discontinuation of treatment, development of macular neovascularization (both eyes), need for rescue treatment, development of ischemic optic neuropathy (both eyes), any serious ocular adverse events (both eyes), and any serious non-ocular adverse events.

For outcomes informing benefit, we will extract outcome data measured within a pre-specified window closest to 1 year (i.e., 44–60 weeks) as well as data from the longest reported follow-up beyond 1 year. For studies that report only short-term outcomes (i.e., follow-up < 44 weeks), we will summarize them separately. For adverse events, we will extract data from the longest reported follow-up available.

For dichotomous outcomes, reviewers, for each arm, will extract the number of participants and events. For continuous outcomes, reviewers, for each arm, will extract the number of participants and mean or median and measure of variability at two timepoints, baseline and end of follow-up, and if available, mean change from baseline and associated measure of variability. We will extract outcome data from the intention-to-treat (ITT) population and, if not reported, in preferred order, modified-ITT, per-protocol (PP), and as-treated population. We will also prioritize outcome data derived from complete-case analysis, when unavailable, in the preferred order: likelihood-based, multiple imputation, and single imputation analyses (e.g., last observation carried forward, worst case/best case).

We anticipate that diagnostic imaging modalities, baseline lesion size, multifocality, lesion location, presence of perilesional hyperautofluorescence, and bilateral involvement might impact the effects of interventions [5]. Thus, we will extract outcome data stratified by these factors, when reported, to facilitate subgroup analyses.

### Risk of bias assessment

For each eligible study and each outcome, paired reviewers, following training and calibration exercises, will independently assess risk of bias and will resolve discrepancies by discussion and, if necessary, by consultation with a third reviewer. We will use the Risk Of Bias instrument for Use in SysTematic reviews – for Randomised Controlled Trials (ROBUST-RCT) [26]. In two steps, reviewers will first assess the implementation of methodological safeguards against biases related to the sequence generation process, allocation concealment, blinding of patients, binding of healthcare providers, blinding of outcome assessors, and missing outcome data. Then, reviewers will judge the risk of bias based on any deficits in the safeguards. We will also evaluate the balance of baseline prognostic factors, validity of outcome measurement for GA lesion area, and termination early for benefit from optional items of the ROBUST-RCT instrument. Reviewers will rate risk of bias as “definitely low risk of bias”, “probably low risk of bias”, “probably high risk of bias”, and “definitely high risk of bias”. When all the domains are low or probably low risk, we will rate the overall risk of bias as “low”; otherwise, we will rate the overall risk of bias as “high”.

### Data synthesis and analysis

To describe data on characteristics of studies, participants, GA lesions, and interventions/comparators, we will use median and interquartile range for continuous variables and frequency and proportions for categorical variables. We will pool dichotomous outcomes using relative risk (RR) and associated 95% confidence interval (CI). To facilitate interpretation of the results for dichotomous outcomes, we will calculate risk difference (RD) per 1000 participants using the baseline risk derived from the median risk of an event across the reference intervention arms (i.e., intervention most connected to others). For continuous outcomes, we will use the change score from baseline to the end of follow-up time to calculate mean difference (MD) and corresponding 95% CI. When authors report outcome data only at baseline and end of follow-up, we will calculate change scores and the associated standard deviation using the median of correlation coefficients derived from trials that reported a change score [27]. If authors only report the end of follow-up score, we will use the follow-up score as a change score and will pool them together [28]. For BCVA and LL-BCVA, we will convert values reported in studies based on Snellen fraction or the logarithm of the minimum angle of resolution to the more intuitive alternative score, the Early Treatment Diabetic Retinopathy Study letter, according to Gregori et al [29].

We will define treatment nodes based on mechanism of action, including, but not limited to, intravitreal complement C3 inhibitors, intravitreal complement C5 inhibitors, intravitreal complement C1q inhibitors, systemic complement inhibitors, regulators of alternative complement pathway, visual cycle modulators, mitochondrial function modulators, individual antioxidants and nutritional supplements, individual gene therapy, regenerative cell-based therapy, trophic cell-based therapy, electrical stimulation, photobiomodulation, individual surgical interventions, and standard care. We will group placebo, sham, and standard care into a single node as “standard care”. We will conduct secondary analyses, separating complement inhibitor nodes by dosing frequency. If we observe differences in treatment effects, we will separate corresponding nodes for the final model.

For each outcome, as long as there are more trials than the number of treatment nodes, we will conduct frequentist random-effects NMA assuming a common heterogeneity across all comparisons under the multivariate meta-analysis models using the restricted maximum likelihood (REML) estimator [30]. If an NMA is not feasible due to disconnected networks or a limited number of studies, we will conduct pairwise meta-analyses for comparisons supported by at least two trials [28]. When quantitative synthesis of evidence is not possible, we will descriptively present results from each included study.

To obtain direct estimates, we will perform pairwise meta-analyses using the REML random-effects model [31]. We will use design-by-treatment models and Wald’s test to assess incoherence at the entire network level [32] and the node-splitting approach to assess incoherence within each closed loop of evidence and to obtain indirect estimates [33]. If important incoherence exists in the network, we will explore potential sources of incoherence considering our a priori defined hypotheses for effect modification: (1) imaging modalities used for diagnosis of GA (FAF vs other techniques) would be associated with different treatment effects; (2) larger versus smaller GA lesion size at baseline would be associated with smaller treatment benefits; (3) multifocal versus unifocal lesions would be associated with smaller treatment benefits; (4) subfoveal versus non-subfoveal lesions would be associated with smaller treatment benefits; (5) presence of perilesional hyperautofluorescence would be associated with smaller treatment benefits; (6) bilateral versus unilateral GA would be associated with smaller treatment benefits; (7) longer versus shorter follow-up time among studies with more than one year of follow-up would be associated with smaller treatment benefits; (8) low versus high risk of bias would be associated with smaller treatment benefits. Network meta-regressions and subgroup analyses will test these hypotheses [34]. We will assess the credibility of any apparent effect modification (p-value for test of interaction < 0.05) using the Instrument to assess the Credibility of Effect Modification Analyses [35].

We will use similar a priori hypotheses to explore potential inconsistency in results between studies informing each direct comparison [36,37]. We will consider the similarity of point estimates, overlap of CIs, I^2^ statistics, and the target of the certainty rating for judgment on inconsistency [37]. If we identify effect modification with moderate to high credibility that explains incoherence or inconsistency, we will conduct separate analyses stratified by subgroups for categorical effect modifiers, and, for continuous variables, we will descriptively present effect modification. If no credible effect modification could explain incoherence or inconsistency, we will rate down the certainty of evidence [37,38].

To assess publication bias, we will use the contour-enhanced funnel plot for each comparison informed by 10 or more studies to visually assess asymmetry in the funnel plots [39] and will test asymmetries in the funnel plots according to Eager et al [40]. We will perform all analyses using the meta and netmeta packages in R (V.4.1.2, Vienna, Austria).

### Assessment of the certainty of the evidence

We will evaluate the certainty of the evidence using the Grading of Recommendations Assessment, Development, and Evaluation (GRADE) framework [41,42] with the target of certainty of a small important effect (i.e., intervention A has a small important effect compared to intervention B) [43]. We will rate the certainty for each comparison and outcome as “high”, “moderate”, “low”, or “very low”. Two methodologists will independently assess each domain, adjudicating disagreements with a third methodologist.

The certainty of evidence for each direct estimate starts as high, and we will rate it down for risk of bias [44], inconsistency [36,37], indirectness [45], and publication bias [46]. For indirect estimates, the certainty starts as the lowest certainty of the contributing direct evidence to the most dominant first-order loop. We will further rate down for intransitivity when important differences exist in the distribution of credible effect modifiers across the direct comparisons informing an indirect estimate [47]. We will assess intransitivity using the same a priori hypotheses for effect modifications specified for exploring incoherence and inconsistency. The certainty of network estimates is based on the direct or indirect evidence that most contributed to the network estimate, with further consideration of rating down for incoherence between direct and indirect estimates [38] and imprecision of network estimates [48]. We will rate down for imprecision if the CI crosses the minimal important difference (MID) threshold. We will decide on MID thresholds for each outcome based on the available literature, and if not available, based on the consensus among clinical experts in the team.

### Interpretation of findings

We will interpret findings using the GRADE approach [49], applying MID thresholds to determine whether the effect of one intervention differs from another. First, if the CI for a comparison between an intervention and the reference crosses the MID, we will classify that intervention in the same group as the reference. If the CI does not cross the MID, we will classify the strategy as more or less effective than the reference, depending on the direction of the effect. Subsequently, among interventions classified as more effective than the reference, we will compare them with one another by examining whether the CI of their estimated effects crosses the MID. Finally, we will categorize interventions based on the certainty of the evidence, restricting our classification to interventions supported by low, moderate, or high certainty evidence relative to the reference.

### Updating and triggers for retirement

This review will be maintained as a living systematic review. Following publication of the initial version, we will conduct updates at six-month intervals to identify and incorporate new evidence as it becomes available. The medical librarian will rerun the search strategy every six months across all prespecified databases. The most recent search for each iteration of the review will be completed no more than three months prior to publication, ensuring that the review remains current while allowing sufficient time for study selection, data extraction, risk of bias assessment and synthesis of evidence.

Adhering to the best practice [50], when no new eligible studies are identified, we will report the date of the last search and the absence of new evidence in our Open Science Framework repository for this review [19]. When new eligible studies are identified, we will extract relevant data, assess risk of bias, and update the analyses.

Every six months, the authors will discuss whether there is added utility to continue with the living systematic review. We will consider retiring this review from the living mode when any of the following conditions are met [51]: 1) high or moderate certainty evidence is achieved across all interventions and outcomes such that additional studies are unlikely to change our confidence in the effect estimates; 2) at least four consecutive updates of the review do not identify new studies or ongoing studies; 3) when clinical experts indicate that the findings are no longer of interest (this criterion might lead to retiring specific interventions or comparators from the living mode); 4) resources are no longer sufficient to sustain the living review.

We will inform evidence users, through Open Science Framework [19], of the status of the review, the anticipated date of the next update, or the decision on terminating the review.

## Discussion

Our living systematic review and network meta-analysis will provide a comprehensive, trustworthy, and up-to-date summary of available evidence on interventions for the management of patients with GA secondary to AMD. This is the first living network meta-analysis investigating the benefits and harms among all pharmacologic and non-pharmacologic interventions for GA. We are not aware of any existing reviews with the same scope and rigor as we propose.

The strengths of this review include a broad eligibility criteria, comprehensive and up-to-date search strategy; duplicate and independent study selection, data extraction, and risk of bias assessment to minimize bias; inclusion of all patient-important outcomes; and application of GRADE methodology to assess the certainty of evidence, interpret findings, and classify interventions from the best to the worst.

This review also has limitations. Despite our comprehensive search strategy, it is possible we will not identify all eligible randomized trials. To mitigate this risk, clinical experts in our team will review the list of eligible RCTs to identify if any important studies are missing. Second, we anticipate that most eligible trials compare active interventions with placebo, sham, or standard care, resulting in sparse direct comparisons between active treatments. Consequently, network estimates comparing active interventions rely primarily on indirect evidence, which may lead to imprecise and uninformative effect estimates. Third, we expect that few studies will contribute to individual medications within the same pharmacologic intervention nodes, limiting our ability to explore treatment effects at the individual drug level. However, we prespecified analyses separating complement inhibitor-related nodes by dosing frequency. Fourth, the GA lesion area is assessed using different imaging modalities with different protocols and analytic approaches. This variability in lesion size measurement may introduce heterogeneity between the results of studies. To address this issue, we will prioritize FAF over other imaging to extract lesion size when available and will summarize untransformed and square root–transformed lesion area separately.

Our findings will support retina specialists in selecting among available interventions for the treatment of GA secondary to AMD and will provide a reliable systematic review of evidence for retina professional societies to inform the development of clinical practice guidelines for GA.

We will disseminate our findings by publication in a peer-reviewed journal and by presentation at national or international conferences. Drawing on the results of our review, we will develop network graphs for each outcome depicting direct and indirect comparisons between different interventions, a colour-coded summary table classifying intervention effects across outcomes, and league tables for each outcome reporting relative and absolute direct, indirect, and network estimates per comparison.

## Supporting information

S1 Table PRISMA-P (Preferred Reporting Items for Systematic review and Meta-Analysis Protocols) 2015 checklist. (DOCX)

S2 File Search strategy. (DOCX)

## References

[1] Flaxman SR, Bourne RRA, Resnikoff S, Ackland P, Braithwaite T, Cicinelli MV, et al. Global causes of blindness and distance vision impairment 1990-2020: a systematic review and meta-analysis. Lancet Glob Health. 2017;5(12):e1221–34. doi: 10.1016/S2214-109X(17)30393-5 29032195

[2] Mitchell P, Liew G, Gopinath B, Wong TY. Age-related macular degeneration. Lancet. 2018;392(10153):1147–59. doi: 10.1016/S0140-6736(18)31550-2 30303083

[3] Holz FG, Strauss EC, Schmitz-Valckenberg S, van Lookeren Campagne M. Geographic atrophy: clinical features and potential therapeutic approaches. Ophthalmology. 2014;121(5):1079–91. doi: 10.1016/j.ophtha.2013.11.023 24433969

[4] McLeod DS, Grebe R, Bhutto I, Merges C, Baba T, Lutty GA. Relationship between RPE and choriocapillaris in age-related macular degeneration. Invest Ophthalmol Vis Sci. 2009;50(10):4982–91. doi: 10.1167/iovs.09-3639 19357355 PMC4829357

[5] Fleckenstein M, Mitchell P, Freund KB, Sadda S, Holz FG, Brittain C, et al. The Progression of Geographic Atrophy Secondary to Age-Related Macular Degeneration. Ophthalmology. 2018;125(3):369–90. doi: 10.1016/j.ophtha.2017.08.038 29110945

[6] Chakravarthy U, Bailey CC, Johnston RL, McKibbin M, Khan RS, Mahmood S, et al. Characterizing Disease Burden and Progression of Geographic Atrophy Secondary to Age-Related Macular Degeneration. Ophthalmology. 2018;125(6):842–9. doi: 10.1016/j.ophtha.2017.11.036 29366564

[7] Taylor DJ, Hobby AE, Binns AM, Crabb DP. How does age-related macular degeneration affect real-world visual ability and quality of life? A systematic review. BMJ Open. 2016;6(12):e011504. doi: 10.1136/bmjopen-2016-011504 27913556 PMC5168634

[8] Woo SJ, Park KH, Ahn J, Choe JY, Jeong H, Han JW, et al. Cognitive impairment in age-related macular degeneration and geographic atrophy. Ophthalmology. 2012;119(10):2094–101. doi: 10.1016/j.ophtha.2012.04.026 22705343

[9] Heier JS, Lad EM, Holz FG, Rosenfeld PJ, Guymer RH, Boyer D, et al. Pegcetacoplan for the treatment of geographic atrophy secondary to age-related macular degeneration (OAKS and DERBY): two multicentre, randomised, double-masked, sham-controlled, phase 3 trials. Lancet. 2023;402(10411):1434–48. doi: 10.1016/S0140-6736(23)01520-9 37865470

[10] Jaffe GJ, Westby K, Csaky KG, Monés J, Pearlman JA, Patel SS, et al. C5 Inhibitor Avacincaptad Pegol for Geographic Atrophy Due to Age-Related Macular Degeneration: A Randomized Pivotal Phase 2/3 Trial. Ophthalmology. 2021;128(4):576–86. doi: 10.1016/j.ophtha.2020.08.027 32882310

[11] Khanani AM, Patel SS, Staurenghi G, Tadayoni R, Danzig CJ, Eichenbaum DA, et al. Efficacy and safety of avacincaptad pegol in patients with geographic atrophy (GATHER2): 12-month results from a randomised, double-masked, phase 3 trial. Lancet. 2023;402(10411):1449–58. doi: 10.1016/S0140-6736(23)01583-0 37696275

[12] Girgis S, Lee LR. Treatment of dry age-related macular degeneration: A review. Clin Exp Ophthalmol. 2023;51(8):835–52. doi: 10.1111/ceo.14294 37737509

[13] Djulbegovic B, Guyatt GH. Progress in evidence-based medicine: a quarter century on. Lancet. 2017;390(10092):415–23. doi: 10.1016/S0140-6736(16)31592-6 28215660

[14] Tzoumas N, Riding G, Williams MA, Steel DH. Complement inhibitors for age-related macular degeneration. Cochrane Database Syst Rev. 2023;6(6):CD009300. doi: 10.1002/14651858.CD009300.pub3 37314061 PMC10266126

[15] Garg A, Nanji K, Tai F, Phillips M, Zeraatkar D, Garg SJ, et al. The effect of complement C3 or C5 inhibition on geographic atrophy secondary to age-related macular degeneration: A living systematic review and meta-analysis. Surv Ophthalmol. 2024;69(3):349–61. doi: 10.1016/j.survophthal.2023.11.008 38008405

[16] Wang H, Zheng J, Zhang Q, Tian Z, Sun Y, Zhu T, et al. Efficacy and safety of complement inhibitors in patients with geographic atrophy associated with age-related macular degeneration: a network meta-analysis of randomized controlled trials. Front Pharmacol. 2024;15:1410172. doi: 10.3389/fphar.2024.1410172 39600369 PMC11589381

[17] Yeong JL, Loveman E, Colquitt JL, Royle P, Waugh N, Lois N. Visual cycle modulators versus placebo or observation for the prevention and treatment of geographic atrophy due to age-related macular degeneration. Cochrane Database Syst Rev. 2020;12(12):CD013154. doi: 10.1002/14651858.CD013154.pub2 33331670 PMC10726984

[18] Moher D, Shamseer L, Clarke M, Ghersi D, Liberati A, Petticrew M, et al. Preferred reporting items for systematic review and meta-analysis protocols (PRISMA-P) 2015 statement. Syst Rev. 2015;4(1):1. doi: 10.1186/2046-4053-4-1 25554246 PMC4320440

[19] Ghadimi M, Begum H, Zeraatkar D, Ma J, Sivaprasad S, Borrelli E, et al. OphthoEvidence Report: Comparative effects of treatments for geographic atrophy secondary to age-related macular degeneration: a protocol for a living systematic review and network meta-analysis. Open Science Framework. 2026. doi: 10.17605/OSF.IO/W4JHR PMC13626355 42814727

[20] Hutton B, Salanti G, Caldwell DM, Chaimani A, Schmid CH, Cameron C, et al. The PRISMA extension statement for reporting of systematic reviews incorporating network meta-analyses of health care interventions: checklist and explanations. Ann Intern Med. 2015;162(11):777–84. doi: 10.7326/M14-2385 26030634

[21] Page MJ, McKenzie JE, Bossuyt PM, Boutron I, Hoffmann TC, Mulrow CD, et al. The PRISMA 2020 statement: an updated guideline for reporting systematic reviews. BMJ. 2021;372:n71. doi: 10.1136/bmj.n71 33782057 PMC8005924

[22] Higgins JP, Eldridge S, Li T. Chapter 23: Including variants on randomized trials [last updated October 2019]. In: Higgins JP, Thomas J, Chandler J, Cumpston M, Li T, Page MJ, et al, editor(s). Cochrane Handbook for Systematic Reviews of Interventions version 6.5. Cochrane, 2024. Available from cochrane.org/handbook [Accessed December 10, 2025].

[23] Haddaway NR, Grainger MJ, Gray CT. Citationchaser: A tool for transparent and efficient forward and backward citation chasing in systematic searching. Res Synth Methods. 2022;13(4):533–45. doi: 10.1002/jrsm.1563 35472127

[24] Covidence systematic review software, Veritas Health Innovation. Melbourne, Australia. Available at www.covidence.org [Accessed December 10, 2025].

[25] Mangione CM, Lee PP, Gutierrez PR, Spritzer K, Berry S, Hays RD, et al. Development of the 25-item National Eye Institute Visual Function Questionnaire. Arch Ophthalmol. 2001;119(7):1050–8. doi: 10.1001/archopht.119.7.1050 11448327

[26] Wang Y, Keitz S, Briel M, Glasziou P, Brignardello-Petersen R, Siemieniuk RAC, et al. Development of ROBUST-RCT: Risk Of Bias instrument for Use in SysTematic reviews-for Randomised Controlled Trials. BMJ. 2025;388:e081199. doi: 10.1136/bmj-2024-081199 40132800

[27] Higgins JP, Li T, Deeks JJ (editors). Chapter 6: Choosing effect measures and computing estimates of effect [last updated August 2023]. In: Higgins JP, Thomas J, Chandler J, Cumpston M, Li T, Page MJ, et al, editor(s). Cochrane Handbook for Systematic Reviews of Interventions version 6.5. Cochrane, 2024. Available from cochrane.org/handbook [Accessed December 12, 2025].

[28] Deeks JJ, Higgins JP, Altman DG, McKenzie JE, Veroniki AA (editors). Chapter 10: Chapter 10: Analysing data and undertaking meta-analyses [last updated November 2024]. In: Higgins JP, Thomas J, Chandler J, Cumpston M, Li T, Page MJ, et al, editor(s). Cochrane Handbook for Systematic Reviews of Interventions version 6.5. Cochrane, 2024. Available from cochrane.org/handbook [Accessed December 12, 2025].

[29] Gregori NZ, Feuer W, Rosenfeld PJ. Novel method for analyzing snellen visual acuity measurements. Retina. 2010;30(7):1046–50. doi: 10.1097/IAE.0b013e3181d87e04 20559157

[30] White IR. Network Meta-analysis. The Stata Journal: Promoting communications on statistics and Stata. 2015;15(4):951–85. doi: 10.1177/1536867x1501500403

[31] Langan D, Higgins JPT, Jackson D, Bowden J, Veroniki AA, Kontopantelis E, et al. A comparison of heterogeneity variance estimators in simulated random-effects meta-analyses. Res Synth Methods. 2019;10(1):83–98. doi: 10.1002/jrsm.1316 30067315

[32] Higgins JPT, Jackson D, Barrett JK, Lu G, Ades AE, White IR. Consistency and inconsistency in network meta-analysis: concepts and models for multi-arm studies. Res Synth Methods. 2012;3(2):98–110. doi: 10.1002/jrsm.1044 26062084 PMC4433772

[33] Dias S, Welton NJ, Caldwell DM, Ades AE. Checking consistency in mixed treatment comparison meta-analysis. Stat Med. 2010;29(7–8):932–44. doi: 10.1002/sim.3767 20213715

[34] White IR, Barrett JK, Jackson D, Higgins JPT. Consistency and inconsistency in network meta-analysis: model estimation using multivariate meta-regression. Res Synth Methods. 2012;3(2):111–25. doi: 10.1002/jrsm.1045 26062085 PMC4433771

[35] Schandelmaier S, Briel M, Varadhan R, Schmid CH, Devasenapathy N, Hayward RA, et al. Development of the Instrument to assess the Credibility of Effect Modification Analyses (ICEMAN) in randomized controlled trials and meta-analyses. CMAJ. 2020;192(32):E901–6. doi: 10.1503/cmaj.200077 32778601 PMC7829020

[36] Guyatt GH, Oxman AD, Kunz R, Woodcock J, Brozek J, Helfand M, et al. GRADE guidelines: 7. Rating the quality of evidence--inconsistency. J Clin Epidemiol. 2011;64(12):1294–302. doi: 10.1016/j.jclinepi.2011.03.017 21803546

[37] Guyatt G, Zhao Y, Mayer M, Briel M, Mustafa R, Izcovich A, et al. GRADE guidance 36: updates to GRADE’s approach to addressing inconsistency. J Clin Epidemiol. 2023;158:70–83. doi: 10.1016/j.jclinepi.2023.03.003 36898507

[38] Brignardello-Petersen R, Mustafa RA, Siemieniuk RAC, Murad MH, Agoritsas T, Izcovich A, et al. GRADE approach to rate the certainty from a network meta-analysis: addressing incoherence. J Clin Epidemiol. 2019;108:77–85. doi: 10.1016/j.jclinepi.2018.11.025 30529648

[39] Sterne JAC, Sutton AJ, Ioannidis JPA, Terrin N, Jones DR, Lau J, et al. Recommendations for examining and interpreting funnel plot asymmetry in meta-analyses of randomised controlled trials. BMJ. 2011;343:d4002. doi: 10.1136/bmj.d4002 21784880

[40] Egger M, Davey Smith G, Schneider M, Minder C. Bias in meta-analysis detected by a simple, graphical test. BMJ. 1997;315(7109):629–34. doi: 10.1136/bmj.315.7109.629 9310563 PMC2127453

[41] Puhan MA, Schünemann HJ, Murad MH, Li T, Brignardello-Petersen R, Singh JA, et al. A GRADE Working Group approach for rating the quality of treatment effect estimates from network meta-analysis. BMJ. 2014;349:g5630. doi: 10.1136/bmj.g5630 25252733

[42] Brignardello-Petersen R, Bonner A, Alexander PE, Siemieniuk RA, Furukawa TA, Rochwerg B, et al. Advances in the GRADE approach to rate the certainty in estimates from a network meta-analysis. J Clin Epidemiol. 2018;93:36–44. 10.1016/j.jclinepi.2017.10.005 29051107

[43] Zeng L, Brignardello-Petersen R, Hultcrantz M, Siemieniuk RAC, Santesso N, Traversy G, et al. GRADE guidelines 32: GRADE offers guidance on choosing targets of GRADE certainty of evidence ratings. J Clin Epidemiol. 2021;137:163–75. doi: 10.1016/j.jclinepi.2021.03.026 33857619

[44] Guyatt GH, Oxman AD, Vist G, Kunz R, Brozek J, Alonso-Coello P, et al. GRADE guidelines: 4. Rating the quality of evidence--study limitations (risk of bias). J Clin Epidemiol. 2011;64(4):407–15. doi: 10.1016/j.jclinepi.2010.07.017 21247734

[45] Guyatt GH, Oxman AD, Kunz R, Woodcock J, Brozek J, Helfand M, et al. GRADE guidelines: 8. Rating the quality of evidence--indirectness. J Clin Epidemiol. 2011;64(12):1303–10. doi: 10.1016/j.jclinepi.2011.04.014 21802903

[46] Guyatt GH, Oxman AD, Montori V, Vist G, Kunz R, Brozek J, et al. GRADE guidelines: 5. Rating the quality of evidence--publication bias. J Clin Epidemiol. 2011;64(12):1277–82. doi: 10.1016/j.jclinepi.2011.01.011 21802904

[47] Brignardello-Petersen R, Tomlinson G, Florez I, Rind DM, Chu D, Morgan R, et al. Grading of recommendations assessment, development, and evaluation concept article 5: addressing intransitivity in a network meta-analysis. J Clin Epidemiol. 2023;160:151–9. doi: 10.1016/j.jclinepi.2023.06.010 37348573

[48] Brignardello-Petersen R, Guyatt GH, Mustafa RA, Chu DK, Hultcrantz M, Schünemann HJ, et al. GRADE guidelines 33: Addressing imprecision in a network meta-analysis. J Clin Epidemiol. 2021;139:49–56. doi: 10.1016/j.jclinepi.2021.07.011 34293434

[49] Brignardello-Petersen R, Florez ID, Izcovich A, Santesso N, Hazlewood G, Alhazanni W, et al. GRADE approach to drawing conclusions from a network meta-analysis using a minimally contextualised framework. BMJ. 2020;371:m3900. doi: 10.1136/bmj.m3900 33177059

[50] Elliott JH, Synnot A, Turner T, Simmonds M, Akl EA, McDonald S, et al. Living systematic review: 1. Introduction-the why, what, when, and how. J Clin Epidemiol. 2017;91:23–30. doi: 10.1016/j.jclinepi.2017.08.010 28912002

[51] Murad MH, Wang Z, Chu H, Lin L, El Mikati IK, Khabsa J, et al. Proposed triggers for retiring a living systematic review. BMJ Evid Based Med. 2023;28(5):348–52. doi: 10.1136/bmjebm-2022-112100 36889900 PMC10579491

