# Supplementary material for: OphthoEvidence Report: Comparative effects of treatments for geographic atrophy secondary to age-related macular degeneration: A protocol for a living systematic review and network meta-analysis
Source: PLoS One. 2026 Sep 30;21(9):e0358699. doi: 10.1371/journal.pone.0358699 (PMC13626355; doi:10.1371/journal.pone.0358699)
Supplement: Supplementary file 2 [file pone.0358699.s002.docx]

**MEDLINE (OVID)**

April 2, 2026

Database: Ovid MEDLINE(R) ALL <1946 to April 01, 2026>

Search Strategy:

--------------------------------------------------------------------------------

1	Geographic Atrophy/ (1376)

2	(geographic adj3 atroph*).mp. (2806)

3	("macular degeneration" and dry).mp. (2157)

4	((late or advanced or dry or age-related or RPE) adj5 ("macular degeneration" or maculopathy or AMD or MD)).mp. (29206)

5	or/1-4 (29737)

6	exp Complement Inactivating Agents/ (12082)

7	exp Complement System Proteins/ (65581)

8	(complement adj3 (cascad* or inhibit* or pathway* or inactivat* or antagon* or modulat* or regulator*)).mp. (29262)

9	((C3 or C5 or C1q) adj3 (inhibit* or antagon* or inactivat*)).mp. (3042)

10	(("factor D" or "factor B" or "factor h") adj3 (inhibit* or antagon* or inactivat*)).mp. (635)

11	(RNA adj3 complement adj3 modulat*).mp. (2)

12	("apl 2" or empaveli* or "avacincaptad pegol" or fabhalta* or danicopan* or izervay* or lampalizumab* or "eculizumab" or lfg316 or iptacopan* or soliris* or compstatin* or tesidolumab* or alxn2040 or apl-2 or apl2 or arc-1905 or arc1905 or aspaveli* or lnp023 or pegcetacoplan* or syfovre* or voydeya* or zimura*).mp. (3905)

13	(pegol adj3 avacincaptad).mp. (68)

14	("ionis fb lrx" or "ionis fblrx" or "isis 696844" or "rg 6299" or "ro 7434656" or ionis-fb-lrx or ionis-fblrx or ionisfblrx or isis696844 or rg6299 or ro7434656 or sefaxersen*).mp. (6)

15	("adx 038" or adx-038 or ngm621 or anx007 or clg561 or al-78898a or "pot 4" or pot4 or avd-104 or "bi 771716" or "gem 103" or aro-cfhrx or "pas nomacopan" or PAS-nomacopan or "cb 2782-peg" or knp301 or knp-301).mp. (23)

16	("apl 3007" or apl-3007 or pozelimab or regn3918 or veopoz or aln-cc5 or cemdisiran).mp. (34)

17	(pozelimab adj3 cemdisiran).mp. (6)

18	("visual cycle" adj3 (modulat* or inhibit* or antagon*)).mp. (63)

19	("visual cycle inhibitor*" or "visual cycle modulator*").mp. (37)

20	deuterated vitamin A.mp. (6)

21	Vitamin A/ or retinol binding protein 4.mp. (27755)

22	gildeuretinol.mp. (0)

23	RBP4.mp. (1549)

24	Retinol-Binding Proteins, Plasma/ai (21)

25	(RBP4 adj3 (modulat* or inhibit* or antagon*)).mp. (58)

26	exp Genetic Therapy/ or gene* therap*.mp. (96244)

27	(gt005 or aavcagscd59 or hmr59 or jnj-1887 or jnj-81201887 or aav5-rora or ocu410 or kriya-825 or sar446597 or ctx001 or pst-611 or pst-611-ct1 or voy-101).mp. (12)

28	Stem Cells/ (73247)

29	exp Stem Cell Transplantation/ (110374)

30	Retinal Pigment Epithelium/ or RPE.mp. (24936)

31	((cell or "stem cell" or cell-based or RPE or "induced pluripotent stem cell" or iPSC-derived) adj3 (therap* or graft* or transplant* or "cell suspension" or allogenic)).mp. (240225)

32	("Bone marrow derived stem cells" or BMSCs).mp. (11548)

33	(ma09-hrpe or cpcb-rpe1 or asp7317 or opregen or rg6501).mp. (10)

34	exp Neuroprotective Agents/ (99859)

35	(mitochondria* adj3 (modulat* or enhanc* or agonist* or therap*)).mp. (17264)

36	(neuroprotect* or neuro-protect*).mp. [mp=title, book title, abstract, original title, name of substance word, subject heading word, floating sub-heading word, keyword heading word, organism supplementary concept word, protocol supplementary concept word, rare disease supplementary concept word, unique identifier, synonyms, population supplementary concept word, anatomy supplementary concept word] (118873)

37	Platelet Activating Factor/ (9231)

38	(platelet* adj2 receptor* inhibit*).mp. (181)

39	PAFR.mp. (384)

40	(bendavia or lamipretide or mtp-131 or gal-101 or mrz-99030 or rt001 or alz-507 or alg-1001 or luminate or risuteganib or brimonidine).mp. (2257)

41	Ciliary Neurotrophic Factor/ or Ciliary neurotrophic factor.mp. (3084)

42	(CNTF or NT-501).mp. (2237)

43	(onl1204 or xelafaslatide or ct1812 or zervimesine or bi 1584862 or brx011 or brx-011).mp. (27)

44	exp Antioxidants/ or antioxidant*.mp. (770718)

45	exp Vitamins/ (372658)

46	vitamin*.mp. (338756)

47	(AREDS or AREDS2).mp. (567)

48	(lutein* or zeaxanthin* or beta-carotene*).mp. (93573)

49	Lutein/ or lutein.mp. (7799)

50	(luteine or xanthophyl or xanthophylls or xantofyl or xantophyl).mp. (6351)

51	zeaxanthin.mp. or Zeaxanthins/ (4798)

52	beta-carotene.mp. or beta Carotene/ (19591)

53	Zinc/ or zinc.mp. (207977)

54	exp Fatty Acids, Omega-3/ or omega-3.mp. (42813)

55	Docosahexaenoic Acids/ or docosahexaenoic.mp. (21167)

56	dhasco.mp. (30)

57	(EG-301 or EG-DPMP-01).mp. (5)

58	Low-Level Light Therapy/ or Photobiomodulation.mp. (10999)

59	(photo-biomodulation or photo-bio-modulation).mp. [mp=title, book title, abstract, original title, name of substance word, subject heading word, floating sub-heading word, keyword heading word, organism supplementary concept word, protocol supplementary concept word, rare disease supplementary concept word, unique identifier, synonyms, population supplementary concept word, anatomy supplementary concept word] (90)

60	("lowlevel light therapy" or "low level light therapy").mp. (9162)

61	valeda.mp. (15)

62	electric* stimulation.mp. or exp Electric Stimulation/ (179265)

63	((retina* or microcurrent or micro-current or trans-corneal or transcorneal or transpalpebral or trans-palpebral) adj3 stimulat*).mp. (2229)

64	TES.mp. (10100)

65	macumira.mp. (1)

66	exp Laser Therapy/ (71817)

67	(((subthreshold or micropulse) adj2 laser) or sml).mp. (1464)

68	OcuDyne.mp. (0)

69	or/6-68 (2350308)

70	5 and 69 (10210)

71	randomized controlled trial.pt. (658312)

72	controlled clinical trial.pt. (95737)

73	randomi?ed.ab. (881274)

74	placebo.ab. (268063)

75	drug therapy.fs. (2920584)

76	randomly.ab. (483690)

77	trial.ab. (810253)

78	groups.ab. (3012821)

79	or/71-78 (6624667)

80	exp animals/ not humans.sh. (5441560)

81	79 not 80 (5819745)

82	70 and 81 (2520)

83	1 or 2 (2806)

84	81 and 83 (882)

85	82 or 84 (2944)

**Embase** <1974 to 2026 March 31>

1	geographic atrophy/	4072

2	(geographic adj3 atroph*).mp.	5052

3	("macular degeneration" and dry).mp.	4185

4	((late or advanced or dry or age-related or RPE) adj5 ("macular degeneration" or maculopathy or AMD or MD)).mp.	50312

5	or/1-4	51337

6	exp complement inhibitor/	4118

7	complement component C3/	30016

8	(complement adj3 (cascad* or inhibit* or pathway* or inactivat* or antagon* or modulat* or regulator*)).mp.	46080

9	stop here.mp.	47

10	((C3 or C5 or C1q) adj3 (inhibit* or antagon* or inactivat*)).mp. 5381

11	(("factor D" or "factor B" or "factor h") adj3 (inhibit* or antagon* or inactivat*)).mp.	1147

12	(RNA adj3 complement adj3 modulat*).mp.	2

13	("apl 2" or empaveli* or "avacincaptad pegol" or fabhalta* or danicopan* or izervay* or lampalizumab* or "eculizumab" or lfg316 or iptacopan* or soliris* or compstatin* or tesidolumab* or alxn2040 or apl-2 or apl2 or arc-1905 or arc1905 or aspaveli* or lnp023 or pegcetacoplan* or syfovre* or voydeya* or zimura*).mp.	14313

14	(pegol adj3 avacincaptad).mp.	325

15	("ionis fb lrx" or "ionis fblrx" or "isis 696844" or "rg 6299" or "ro 7434656" or ionis-fb-lrx or ionis-fblrx or ionisfblrx or isis696844 or rg6299 or ro7434656 or sefaxersen*).mp.	48

16	("adx 038" or adx-038 or ngm621 or anx007 or clg561 or al-78898a or "pot 4" or pot4 or avd-104 or "bi 771716" or "gem 103" or aro-cfhrx or "pas nomacopan" or PAS-nomacopan or "cb 2782-peg" or knp301 or knp-301).mp.	144

17	("apl 3007" or apl-3007 or pozelimab or regn3918 or veopoz or aln-cc5 or cemdisiran).mp.	213

18	(pozelimab adj3 cemdisiran).mp.	35

19	("visual cycle" adj3 (modulat* or inhibit* or antagon*)).mp. 106

20	("visual cycle inhibitor*" or "visual cycle modulator*").mp.	62

21	deuterated vitamin A.mp.	10

22	retinol binding protein 4/	2875

23	gildeuretinol.mp. or gildeuretinol/	40

24	RBP4.mp.	1991

25	plasma retinol binding protein/	240

26	exp gene therapy/	117001

27	gene* therap*.mp.	143363

28	(gt005 or aavcagscd59 or hmr59 or jnj-1887 or jnj-81201887 or aav5-rora or ocu410 or kriya-825 or sar446597 or ctx001 or pst-611 or pst-611-ct1 or voy-101).mp.	89

29	exp stem cell/	564831

30	exp stem cell transplantation/	229892

31	RPE-340 cell line/ or RPE.mp. or retinal pigment epithelium/	39284

32	((cell or "stem cell" or cell-based or RPE or "induced pluripotent stem cell" or iPSC-derived) adj3 (therap* or graft* or transplant* or "cell suspension" or allogenic)).mp.	470285

33	("Bone marrow derived stem cells" or BMSCs).mp.	15232

34	(ma09-hrpe or cpcb-rpe1 or asp7317 or opregen or rg6501).mp.	39

35	exp neuroprotective agent/	380037

36	(mitochondria* adj3 (modulat* or enhanc* or agonist* or therap*)).mp. 22066

37	(neuroprotect* or neuro-protect*).mp.	184512

38	thrombocyte activating factor/	15437

39	(platelet* adj2 receptor* inhibit*).mp.	286

40	PAFR.mp.	526

41	(bendavia or lamipretide or mtp-131 or gal-101 or mrz-99030 or rt001 or alz-507 or alg-1001 or luminate or risuteganib or brimonidine).mp.	7496

42	Ciliary neurotrophic factor.mp. or ciliary neurotrophic factor/	5463

43	(CNTF or NT-501).mp.	2874

44	(onl1204 or xelafaslatide or ct1812 or zervimesine or bi 1584862 or brx011 or brx-011).mp.	92

45	exp antioxidant/ or antioxidant*.mp.	816403

46	exp vitamin/	862845

47	vitamin*.mp.	468260

48	(AREDS or AREDS2).mp.	1072

49	(lutein* or zeaxanthin* or beta-carotene*).mp.	138023

50	xanthophyll/ or lutein.mp.	13364

51	(luteine or xanthophyl or xanthophylls or xantofyl or xantophyl).mp. 1352

52	zeaxanthin*.mp. or zeaxanthin/	7112

53	beta-carotene.mp. or beta carotene/	30701

54	zinc/ or zinc.mp.	340311

55	omega 3.mp. or exp omega 3 fatty acid/	56010

56	docosahexaenoic acid/ or docosahexaenoic.mp.	34935

57	dhasco.mp.	70

58	(EG-301 or EG-DPMP-01).mp. [mp=title, abstract, heading word, drug trade name, original title, device manufacturer, drug manufacturer, device trade name, keyword heading word, floating subheading word, candidate term word]	10

59	Photobiomodulation.mp. or low level laser therapy/	32622

60	(photo-biomodulation or photo-bio-modulation).mp.	137

61	("lowlevel light therapy" or "low level light therapy").mp.	887

62	valeda.mp.	35

63	electric* stimulation.mp. or electrostimulation/	123310

64	((retina* or microcurrent or micro-current or trans-corneal or transcorneal or transpalpebral or trans-palpebral) adj3 stimulat*).mp. 2989

65	TES.mp.	12556

66	macumira.mp.	1

67	exp laser therapy/	38984

68	(((subthreshold or micropulse) adj2 laser) or sml).mp.	2161

69	OcuDyne.mp.	8

70	or/6-69	3709075

71	5 and 70	18684

72	randomized controlled trial/	1155373

73	Controlled clinical study/	461809

74	random$.ti,ab.	2611938

75	randomization/	102066

76	intermethod comparison/	320048

77	placebo.ti,ab.	479346

78	(compare or compared or comparison).ti.	710121

79	((evaluated or evaluate or evaluating or assessed or assess) and (compare or compared or comparing or comparison)).ab.	3528169

80	(open adj label).ti,ab.	197609

81	((double or single or doubly or singly) adj (blind or blinded or blindly)).ti,ab.	379318

82	double blind procedure/	320530

83	parallel group$1.ti,ab.	54969

84	(crossover or cross over).ti,ab.	161342

85	((assign$ or match or matched or allocation) adj5 (alternate or group$1 or intervention$1 or patient$1 or subject$1 or participant$1)).ti,ab. 535863

86	(assigned or allocated).ti,ab.	636687

87	(controlled adj7 (study or design or trial)).ti,ab.	665140

88	(volunteer or volunteers).ti,ab.	329670

89	human experiment/	750196

90	trial.ti.	591081

91	or/72-90	7842246

92	(random$ adj sampl$ adj7 ("cross section$" or questionnaire$1 or survey$ or database$1)).ti,ab. not (comparative study/ or controlled study/ or randomi?ed controlled.ti,ab. or randomly assigned.ti,ab.)	11008

93	Cross-sectional study/ not (randomized controlled trial/ or controlled clinical study/ or controlled study/ or randomi?ed controlled.ti,ab. or control group$1.ti,ab.)	503956

94	(((case adj control$) and random$) not randomi?ed controlled).ti,ab. 25438

95	(Systematic review not (trial or study)).ti.	371785

96	(nonrandom$ not random$).ti,ab.	21820

97	"Random field$".ti,ab.	3335

98	(random cluster adj3 sampl$).ti,ab.	1835

99	(review.ab. and review.pt.) not trial.ti.	1392886

100	"we searched".ab. and (review.ti. or review.pt.) 63255

101	"update review".ab.	156

102	(databases adj4 searched).ab.	84151

103	(rat or rats or mouse or mice or swine or porcine or murine or sheep or lambs or pigs or piglets or rabbit or rabbits or cat or cats or dog or dogs or cattle or bovine or monkey or monkeys or trout or marmoset$1).ti. and animal experiment/	1346433

104	Animal experiment/ not (human experiment/ or human/)	2853802

105	or/92-104	5143066

106	91 not 105	6910159

107	71 and 106	3332

108	1 or 2	5052

109	106 and 108	1549

110	107 or 109	4052

**Cochrane** (Wiley)

Search Name:	2026-04 Geographic atrophy

Date Run:	02/04/2026 18:33:16

Comment:

ID	Search	Hits

#1	MeSH descriptor: [Geographic Atrophy] explode all trees	225

#2	(geographic NEAR/3 atroph*)	600

#3	("macular degeneration" and dry)	343

#4	((late or advanced or dry or age-related or RPE) NEAR/5 ("macular degeneration" or maculopathy or AMD or MD))	3959

#5	#1 or #2 or #3 or #4	4041

#6	MeSH descriptor: [Complement Inactivating Agents] explode all trees 132

#7	MeSH descriptor: [Complement System Proteins] explode all trees	906

#8	(complement NEAR/3 (cascad* or inhibit* or pathway* or inactivat* or antagon* or modulat* or regulator*))	1301

#9	((C3 or C5 or C1q) NEAR/3 (inhibit* or antagon* or inactivat*))	448

#10	(("factor D" or "factor B" or "factor h") NEAR/3 (inhibit* or antagon* or inactivat*))	125

#11	(RNA NEAR/3 complement NEAR/3 modulat*)	0

#12	("apl 2" or empaveli* or "avacincaptad pegol" or fabhalta* or danicopan* or izervay* or lampalizumab* or "eculizumab" or lfg316 or iptacopan* or soliris* or compstatin* or tesidolumab* or alxn2040 or apl-2 or apl2 or arc-1905 or arc1905 or aspaveli* or lnp023 or pegcetacoplan* or syfovre* or voydeya* or zimura*)	955

#13	(pegol NEAR/3 avacincaptad)	33

#14	("ionis fb lrx" or "ionis fblrx" or "isis 696844" or "rg 6299" or "ro 7434656" or ionis-fb-lrx or ionis-fblrx or ionisfblrx or isis696844 or rg6299 or ro7434656 or sefaxersen*)	15

#15	("adx 038" or adx-038 or ngm621 or anx007 or clg561 or al-78898a or "pot 4" or pot4 or avd-104 or "bi 771716" or "gem 103" or aro-cfhrx or "pas nomacopan" or PAS-nomacopan or "cb 2782-peg" or knp301 or knp-301)	23

#16	("apl 3007" or apl-3007 or pozelimab or regn3918 or veopoz or aln-cc5 or cemdisiran)	47

#17	(pozelimab NEAR/3 cemdisiran)	24

#18	("visual cycle" NEAR/3 (modulat* or inhibit* or antagon*))	14

#19	("visual cycle inhibitor" or "visual cycle modulator")	9

#20	deuterated vitamin A	20

#21	retinol binding protein 4	481

#22	MeSH descriptor: [Vitamin A] explode all trees	2546

#23	gildeuretinol	4

#24	RBP4	93

#25	MeSH descriptor: [Retinol-Binding Proteins, Plasma] explode all trees and with qualifier(s): [antagonists & inhibitors - AI]	2

#26	MeSH descriptor: [Genetic Therapy] explode all trees	390

#27	gene* therap*	204804

#28	gt005 or aavcagscd59 or hmr59 or jnj1887 or jnj81201887 or aav5rora or ocu410 or kriya825 or sar446597 or ctx001 or pst611 or pst611ct1 or voy101	14

#29	MeSH descriptor: [Stem Cells] explode all trees	1360

#30	MeSH descriptor: [Stem Cell Transplantation] explode all trees	3658

#31	MeSH descriptor: [Retinal Pigment Epithelium] explode all trees	96

#32	RPE	3227

#33	(cell or "stem cell" or cell-based or RPE or "induced pluripotent stem cell" or iPSC-derived) NEAR/3 (therap* or graft* or transplant* or "cell suspension" or allogenic)	24229

#34	"Bone marrow derived stem cells" or BMSCs	190

#35	ma09hrpe or cpcbrpe1 or asp7317 or opregen or rg6501	5

#36	MeSH descriptor: [Neuroprotective Agents] explode all trees	1302

#37	(mitochondria* NEAR/3 (modulat* or enhanc* or agonist* or therap*)) 378

#38	(neuroprotect* or neuro-protect*)	5759

#39	MeSH descriptor: [Platelet Activating Factor] explode all trees	179

#40	(platelet* NEAR/2 receptor* inhibit*)	363

#41	PAFR	6

#42	(bendavia or lamipretide or mtp-131 or gal-101 or mrz-99030 or rt001 or alz-507 or alg-1001 or luminate or risuteganib or brimonidine)	881

#43	MeSH descriptor: [Ciliary Neurotrophic Factor] explode all trees	24

#44	Ciliary Neurotrophic Factor	72

#45	(CNTF or NT-501)	65

#46	(onl1204 or xelafaslatide or ct1812 or zervimesine or bi 1584862 or brx011 or brx-011)	56

#47	MeSH descriptor: [Antioxidants] explode all trees	6455

#48	antioxidant*	18741

#49	MeSH descriptor: [Vitamins] explode all trees	5738

#50	vitamin*	42434

#51	(AREDS or AREDS2)	293

#52	(lutein* or zeaxanthin* or beta-carotene*)	7526

#53	MeSH descriptor: [Luteinization] explode all trees	31

#54	(luteine or xanthophyl or xanthophylls or xantofyl or xantophyl) 240

#55	MeSH descriptor: [Zeaxanthins] explode all trees	169

#56	Zeaxanthin*	567

#57	MeSH descriptor: [beta Carotene] explode all trees	921

#58	MeSH descriptor: [Zinc] explode all trees	2044

#59	zinc	8017

#60	MeSH descriptor: [Fatty Acids, Omega-3] explode all trees	4311

#61	omega-3	7733

#62	MeSH descriptor: [Docosahexaenoic Acids] explode all trees	1624

#63	Docosahexaenoic	3706

#64	dhasco	10

#65	(EG-301 or EG-DPMP-01)	1

#66	MeSH descriptor: [Low-Level Light Therapy] explode all trees	1884

#67	Photobiomodulation	1944

#68	(photo-biomodulation or photo-bio-modulation)	65

#69	("lowlevel light therapy" or "low level light therapy")	1978

#70	valeda	9

#71	MeSH descriptor: [Electric Stimulation] explode all trees	2634

#72	electric* stimulation	20252

#73	((retina* or microcurrent or micro-current or trans-corneal or transcorneal or transpalpebral or trans-palpebral) NEAR/3 stimulat*)	250

#74	TES	886

#75	macumira	1

#76	MeSH descriptor: [Laser Therapy] explode all trees	6283

#77	(((subthreshold or micropulse) NEAR/2 laser) or sml)	316

#78	OcuDyne	0

#79	6 or #7 or #8 or #9 or #10 or #11 or #12 or #13 or #14 or #15 or #16 or #17 or #18 or #19 or #20 or #21 or #22 or #23 or #24 or #25 or #26 or #27 or #28 or #29 or #30 or #31 or #32 or #33 or #34 or #35 or #36 or #37 or #38 or #39 or #40 or #41 or #42 or #43 or #44 or #45 or #46 or #47 or #48 or #49 or #50 or #51 or #52 or #53 or #54 or #55 or #56 or #57 or #58 or #59 or #60 or #61 or #62 or #63 or #64 or #65 or #66 or #67 or #68 or #69 or #70 or #71 or #72 or #73 or #74 or #75 or #76 or #77 or #78	1092552

#80	#5 and #79 in Trials	2538

#81	#1 or #2 in Trials	574

#82	#80 or #81	2630

**Web of Science** (Clarivate)

3

#1 AND #2

256

2

TS=(randomised OR randomized OR randomisation OR randomization OR placebo* OR (random* AND (allocat* OR assign*) ) OR (blind* AND (single OR double OR treble OR triple) )) NOT TS=(animal or animals or pisces or fish or fishes or catfish or catfishes or sheatfish or silurus or arius or heteropneustes or clarias or gariepinus or fathead minnow or fathead minnows or pimephales or promelas or cichlidae or trout or trouts or char or chars or salvelinus or salmo or oncorhynchus or guppy or guppies or millionfish or poecilia or goldfish or goldfishes or carassius or auratus or mullet or mullets or mugil or curema or shark or sharks or cod or cods or gadus or morhua or carp or carps or cyprinus or carpio or killifish or eel or eels or anguilla or zander or sander or lucioperca or stizostedion or turbot or turbots or psetta or flatfish or flatfishes or plaice or pleuronectes or platessa or tilapia or tilapias or oreochromis or sarotherodon or common sole or dover sole or solea or zebrafish or zebrafishes or danio or rerio or seabass or dicentrarchus or labrax or morone or lamprey or lampreys or petromyzon or pumpkinseed or pumpkinseeds or lepomis or gibbosus or herring or clupea or harengus or amphibia or amphibian or amphibians or anura or salientia or frog or frogs or rana or toad or toads or bufo or xenopus or laevis or bombina or epidalea or calamita or salamander or salamanders or newt or newts or triturus or reptilia or reptile or reptiles or bearded dragon or pogona or vitticeps or iguana or iguanas or lizard or lizards or anguis fragilis or turtle or turtles or snakes or snake or aves or bird or birds or quail or quails or coturnix or bobwhite or colinus or virginianus or poultry or poultries or fowl or fowls or chicken or chickens or gallus or zebra finch or taeniopygia or guttata or canary or canaries or serinus or canaria or parakeet or parakeets or grasskeet or parrot or parrots or psittacine or psittacines or shelduck or tadorna or goose or geese or branta or leucopsis or woodlark or lullula or flycatcher or ficedula or hypoleuca or dove or doves or geopelia or cuneata or duck or ducks or greylag or graylag or anser or harrier or circus pygargus or red knot or great knot or calidris or canutus or godwit or limosa or lapponica or meleagris or gallopavo or jackdaw or corvus or monedula or ruff or philomachus or pugnax or lapwing or peewit or plover or vanellus or swan or cygnus or columbianus or bewickii or gull or chroicocephalus or ridibundus or albifrons or great tit or parus or aythya or fuligula or streptopelia or risoria or spoonbill or platalea or leucorodia or blackbird or turdus or merula or blue tit or cyanistes or pigeon or pigeons or columba or pintail or anas or starling or sturnus or owl or athene noctua or pochard or ferina or cockatiel or nymphicus or hollandicus or skylark or alauda or tern or sterna or teal or crecca or oystercatcher or haematopus or ostralegus or shrew or shrews or sorex or araneus or crocidura or russula or european mole or talpa or chiroptera or bat or bats or eptesicus or serotinus or myotis or dasycneme or daubentonii or pipistrelle or pipistrellus or cat or cats or felis or catus or feline or dog or dogs or canis or canine or canines or otter or otters or lutra or badger or badgers or meles or fitchew or fitch or foumart or foulmart or ferrets or ferret or polecat or polecats or mustela or putorius or weasel or weasels or fox or foxes or vulpes or common seal or phoca or vitulina or grey seal or halichoerus or horse or horses or equus or equine or equidae or donkey or donkeys or mule or mules or pig or pigs or swine or swines or hog or hogs or boar or boars or porcine or piglet or piglets or sus or scrofa or llama or llamas or lama or glama or deer or deers or cervus or elaphus or cow or cows or bos taurus or bos indicus or bovine or bull or bulls or cattle or bison or bisons or sheep or sheeps or ovis aries or ovine or lamb or lambs or mouflon or mouflons or goat or goats or capra or caprine or chamois or rupicapra or leporidae or lagomorpha or lagomorph or rabbit or rabbits or oryctolagus or cuniculus or laprine or hares or lepus or rodentia or rodent or rodents or murinae or mouse or mice or mus or musculus or murine or woodmouse or apodemus or rat or rats or rattus or norvegicus or guinea pig or guinea pigs or cavia or porcellus or hamster or hamsters or mesocricetus or cricetulus or cricetus or gerbil or gerbils or jird or jirds or meriones or unguiculatus or jerboa or jerboas or jaculus or chinchilla or chinchillas or beaver or beavers or castor fiber or castor canadensis or sciuridae or squirrel or squirrels or sciurus or chipmunk or chipmunks or marmot or marmots or marmota or suslik or susliks or spermophilus or cynomys or cottonrat or cottonrats or sigmodon or vole or voles or microtus or myodes or glareolus or primate or primates or prosimian or prosimians or lemur or lemurs or lemuridae or loris or bush baby or bush babies or bushbaby or bushbabies or galago or galagos or anthropoidea or anthropoids or simian or simians or monkey or monkeys or marmoset or marmosets or callithrix or cebuella or tamarin or tamarins or saguinus or leontopithecus or squirrel monkey or squirrel monkeys or saimiri or night monkey or night monkeys or owl monkey or owl monkeys or douroucoulis or aotus or spider monkey or spider monkeys or ateles or baboon or baboons or papio or rhesus monkey or macaque or macaca or mulatta or cynomolgus or fascicularis or green monkey or green monkeys or chlorocebus or vervet or vervets or pygerythrus or hominoidea or ape or apes or hylobatidae or gibbon or gibbons or siamang or siamangs or nomascus or symphalangus or hominidae or orangutan or orangutans or pongo or chimpanzee or chimpanzees or pan troglodytes or bonobo or bonobos or pan paniscus or gorilla or gorillas or troglodytes)

1,647,008

1

geographic atrophy (Topic)

4,635

ClinicalTrial.gov

Geographic atrophy
